# Age estimation from the immature pars lateralis using decision tree analysis

**DOI:** 10.1007/s00414-026-03788-z

**Published:** 2026-04-10

**Authors:** Deona Botha, Erin F. Hutchinson, Roxanne Thornton

**Affiliations:** 1 Human Variation and Identification Research Unit, Department of Anatomical Sciences, Faculty of Health Sciences, University of the Witwatersrand, 7 York Road, Parktown, Johannesburg, South Africa; 2 Department of Forensic Medicine and Pathology, Faculty of Health Sciences, University of the Witwatersrand, 7 York Road, Parktown, Johannesburg, South Africa

**Keywords:** pars lateralis, Fetal osteology, skeletal age, machine learning, decision trees

## Abstract

Age-at-death estimation is instrumental to the medicolegal investigation of unknown fetal and infant remains due to a large number of such remains received by medico-legal laboratories in South Africa. The maturation and growth of immature bones serves as an alternative to soft tissue and dental age estimation. The development of bones such as the pars lateralis serves as one of the possible ways to estimate age in immature individuals. The aim of this study was to test previously described osteomorphological descriptors of the pars lateralis associated with early prenatal, late prenatal and early postnatal development in relation to estimating age. Data from a sample of 99 human pars laterali originating from the Johannesburg Forensic Paediatric Collection (JFPC), University of the Witwatersrand was utilized in the construction of decision trees with the use of the WEKA (version 3.9.3) software for the purposes of forensic age estimation of skeletal remains. The study sample was subdivided into early prenatal (younger than 30 gestational weeks; *n* = 29), late prenatal (30–40 gestational weeks, *n* = 40) and early postnatal (birth to 7.5 months, *n* = 30) age groups. A pruned tree algorithm (J48) was used to construct five decision trees and included four morphological variables associated with development the pars lateralis. Overall, 85.9% of all individuals were correctly classified using the combination of all four variables. Features associated with specific areas on the bone displayed a range of classification success (jugular limb – 78.8%; shape of bone – 75.8%; medial border of foramen magnum – 64.7%; hypoglossal canal – 63.6%). This study highlights the supportive role of the pars lateralis for forensic age estimation. Furthermore, machine learning algorithms provide quantitative confirmation of proposed morphological age predictors applicable to biological profiling of immature remains.

## Introduction

Biological profiling of Fetal and infant human remains is predominately reliant on age estimation. A fundamental aim in the medico-legal investigation of Fetal and infant remains is the establishment of separate existence and viability of individuals which within the South African legal framework is essential for potential prosecution in cases where foul play is suspected [1, 2]. The skull is useful in anthropometric age estimations, particularly in the absence of dentition and the appendicular skeleton [3]. Rapid and dynamic maturation of the skull base, owing to its sequential pattern of ossification, may aid forensic identification of unclaimed and unidentified Fetal and infant individuals [1, 4]. The development of the basicranium has received some attention, due to its maturation sequence and contribution of components to the border of the foramen magnum during pre- and post-partum development, but thus far limited detailed investigation has been extended to the individual components of this region and its potential application in age estimation [5–7].

The developing pars squama, pars laterali and pars basilaris form the immature occipital complex [8, 9]. While the pars basilaris and pars squama have been studied [1, 7, 10–13], the pars lateralis is relatively understudied for application to forensic age estimation, despite this bone being resilient to taphonomic factors and often recovered in forensic contexts [4]. Located in the posterior cranial fossa, the pars lateralis is important anatomically due to its close relationship with surrounding structures such as the pars temporalis complex, pars basilaris, pars squama, posterior intra-occipital synchondrosis (PIOS) and anterior intra-occipital synchondrosis (AIOS) [5, 6]. The pars lateralis also provides stability for the developing cerebellum and transition of neurovascular structures [14]. The developmental importance of the pars lateralis and probability of its recovery support the use of the pars lateralis in routine case work and disaster victim scenarios. Thus, an understanding of the development and growth of the pars lateralis is essential for including the structural and functional role of the basicranium within a forensic setting.

Studies on the pars lateralis are limited to quantitative anthropometric studies, which are primarily focused on changes in the dimension of the bone [1, 2, 12]. Anthropometric indices based on 20th century Hungarian remains aged between 12 gestational weeks and birth [12] document age related changes in the maximum length and width of the pars lateralis during the Fetal period. Additionally, these dimensional changes have been observed in a modern South African sample of early postnatal (birth to 7.5 months) individuals [1]. Figueiro et al. [2] investigated the applicability of additional measurement criteria of the anterior synchondrosis and occipital condyle for age at death estimation. A sample of 127 individuals aged between 6 months in utero and 4 years postnatal were subject to regression functions for age estimation. Functions derived from these measurements are applicable to recovered incomplete pars laterali from individuals up to 2 years of age [2].

While quantitative methods have proven useful in the forensic context thus far, morphological descriptors and criteria of unfused skeletal elements may also prove useful. This is relevant when differentiating between bones of similar morphology, such as the immature scapula and the pars lateralis [7]. During the perinatal phase, the intracranial surface of the pars lateralis is similar in morphology as the dorsal surface of the scapula. In addition, depending on the orientation of the bone, the jugular limb could be mistaken for the scapular spine. However, despite these similarities in shape, the overall size of the scapular blade is a discriminating factor. Furthermore, identification of the immature hypoglossal canal distinquishes this skeletal element as part of head and neck anatomy instead of a component of the pectoral girdle.

Identification of the unfused pars lateralis is further confounded by environmental factors. Taphonomic conditions can alter the postmortem composition of bone and therefore influence the resilience of newly formed osseous tissue [15] which influences proper recovery of remains. Previous work on morphological changes associated with age intervals, include limited morphological descriptions during the Fetal period and stages of fusion which occur in the postnatal stage of development [5, 6, 16]. Redfield’s (1970) investigation on a limited sample of Fetal and infant archaeological remains provided an invaluable resource on the size of the pars lateralis relative to the pars basilaris during the Fetal and infant period. In addition, brief descriptions of the hypoglossal canal and anterior and posterior synchondrosis were included in the stages of development for the Fetal and postnatal period of growth [16]. The development of the hypoglossal canal has subsequently been investigated with a focus on the closure of synchondroses being associated with the age and the shape of the foramen magnum when examining maturation of 152 skulls of European origin [5]. Similarly, data generated from 64 postnatal individuals on the stages of fusion between the condylar and jugular limbs which form the hypoglossal canal, indicates that the PIOS fuses earlier than the AIOS [6]. These studies contribute to the knowledge base concerning the growth and subsequent development of the adult derivative, but negate the morphology and maturation of specific regions, which are instrumental to neurodevelopment and proper recovery of Fetal and infant skeletal fragments. Recently, age estimating criteria utilizing morphological predictors based on the immature pars lateralis have been proposed [14]. Age related changes during the early prenatal, prenatal, and postnatal stages of development (*n* = 101) were identified from unidentified and unclaimed forensic fetal remains. Morphological criteria was based on the bone shape, ossification patterning, medial and mastoid temporal border morphology, and development of the hypoglossal canal [4, 25]. Examination of these surfaces and borders indicated changes in shape and size during in utero development and the first year of life, which suggest accommodation for the developing brain and adjacent soft tissues. Application of this data set to statistical models is necessary to test the validity and use of morphological predictors in forensic case work.

Traditional classification methods for age estimation employed in constructing a biological profile typically rely on qualitative methods derived from anatomical features [17]. To mitigate the subjectivity of qualitative observations and provide objectivity and reliability, employment of statistical methods to quantify classification techniques may be used. Decision tree analysis as a form of machine learning has been incorporated into study designs to mitigate the subjectivity bias and challenges related to morphometric analyses [18]. Decision tree analyses have been applied and evaluated for sex estimation of the femur and non-metric cranial traits [19, 20], ancestry from cranial anthropometric data [21, 22], as well as age estimation utilizing the sternal rib end, auricular surface, and pubic symphysis [23, 24]. Machine learning approaches have been employed for various parameters of biological profiling, with promising results for future method development [23]. The application of machine learning methods such as decision tree analysis using the juvenile skeleton should thus also be considered. The aim of this study was to apply decision tree analysis as a machine learning approach for age classification in Fetal and infant skeletal remains using morphological age indicators of the pars lateralis as an alternative approach to traditional methods.

## Materials and methods

The sample used in this study originated from the Johannesburg Forensic Paediatric Collection (JFPC), which comprises of individuals of unknown provenance [14]. The JFPC is an ongoing humanitarian and scientific initiative, which aims to preserve and safeguard unidentified immature skeletal remains. Individuals included in the JFPC are unclaimed and unidentified decedents previously admitted to the Forensic Pathology Services for medicolegal examination. Under the National Health Act, 2003 (Act No. 61 of 2003 [26], the function of the Forensic Pathology Service in the Department of Health (DOH) in South Africa is the medico-legal investigation of unnatural death. Fetal and infant remains are admitted to the services in accordance with the Births and Deaths Registration Act no.51 of 1992, Sect. 12 [27], and the Criminal Procedure Act, Sect. 239 [28].

Individuals included in the sample were previously aged at autopsy with standard anthropometric measurements in accordance with the Guihard-Costa method [29]. This method includes standardized fetal body measurements such as Crown-heel length, Crown-rump Length and abdominal circumferences, which are routinely followed during post mortem examination at the Johannesburg Medico-Legal Laboratory. Following inclusion into the JFPC, individuals were further aged using dental [30] or fundamental anthropometric skeletal aging criteria by Fazekas and Kosa [12, 31]. Biological sex was assigned at autopsy or via molecular assay [32].

A total sample of 99 immature crania previously evaluated in terms of morphological features associated with age at death [7, 14], were analysed using decision tree analysis to extract age-related patterns in the data. The study sample was subdivided into early prenatal (30 gestational weeks, *n* = 29), late prenatal (30 to 40 gestational weeks *n* = 40) and early postnatal (birth to 7.5 months, *n* = 30) groups respectively.

**Fig. 1:**
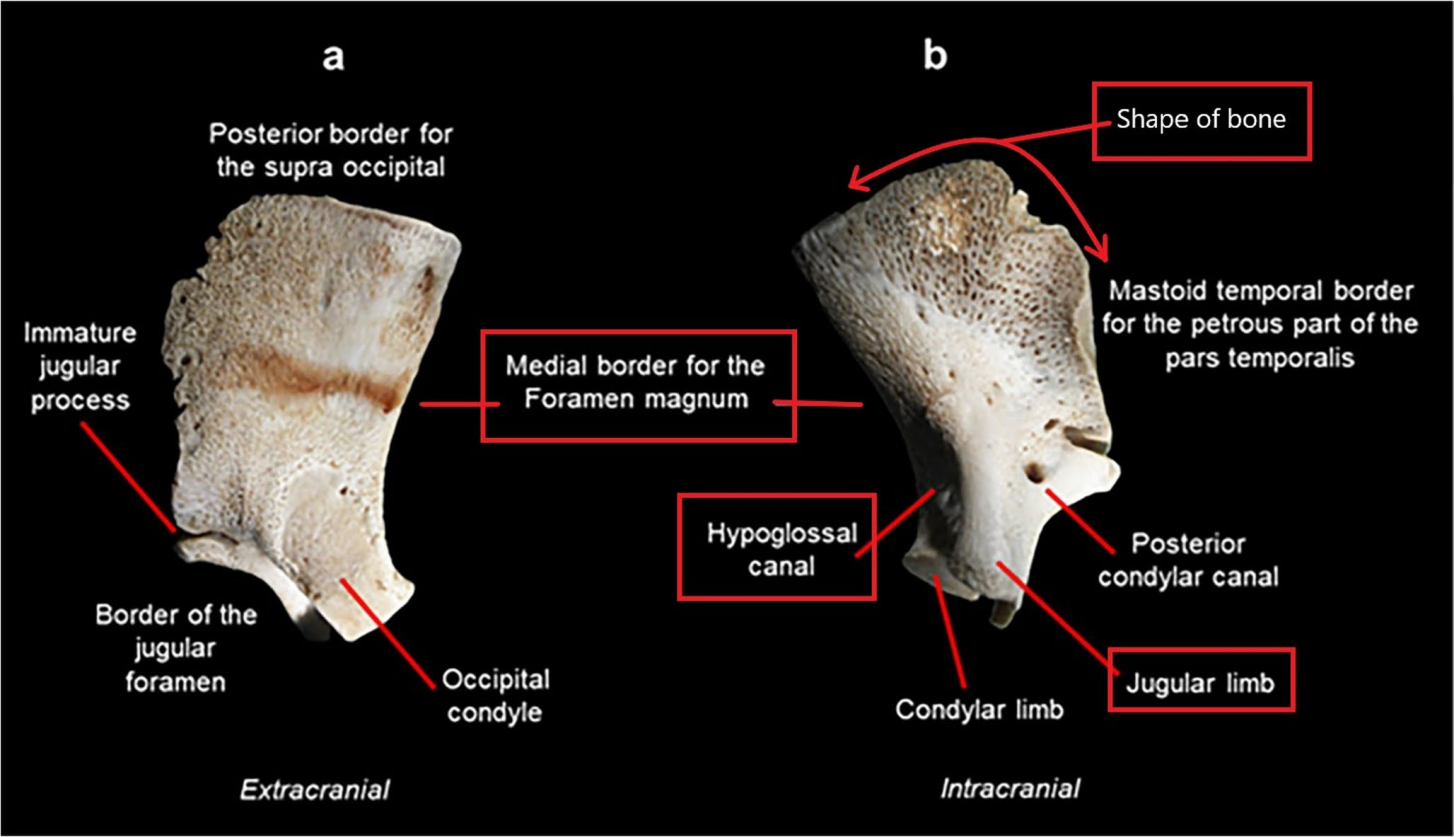
Prenatal pars lateralis representing relevant osteological features. The regions used in this study are boxed in red. (**a**) Extracranial view of the left pars lateralis representing posterior border, immature jugular process, border of the jugular foramen, occipital condyle, and medial border for the foramen magnum. (**b**) Intracranial view of the left pars lateralis representing mastoid temporal border for the petrous part of the pars temporalis, posterior condylar canal, jugular and condylar limb contributing the hypoglossal canal and medial border for the foramen magnum. Magnification (1.5–2.5 ×) performed on Nikon Stereomicroscope (SMZ1500, Japan)

Morphological characteristics associated with four areas on the bone were assessed and included features related to the overall shape of the bone, medial border of the foramen magnum, the jugular limb, and hypoglossal canal of the pars lateralis (Fig. 1; Table 1). Characteristics are listed in the order in which it develop during the early prenatal, late prenatal and early postnatal stages of development [14]. 

**Table 1:** Morphological features associated with various regions of the pars lateralis indicative of age. (adapted from Thornton et al. [14])*.

Region and feature	Description of feature		Stage of Development
Shape of bone			
SB1		Triangular	Early Prenatal
SB2		Quadrilateral	Late prenatal
**Medial border of Foramen Magnum**			
FM1		Straight	Early Prenatal
FM2		Curved	Late prenatal
FM3		V-shaped	Early postnatal
**Jugular limb**			
JL1		Presence of oval eminence	Late prenatal
JL2		Straight and thickened protuberance	Late prenatal
JL3		Hooking of articular facet	Early postnatal
**Hypoglossal canal**			
HC1		Straight and thickened	Early prenatal
HC2		Medial curvature	Late prenatal
HC3		Condylar limb shorter than jugular limb	Late prenatal

*Refer to Fig. 1 for an illustration of skeletal anatomy associated with the immature pars lateralis

Ethical approval was granted through the Human Research Ethics Committee – Medical of the University of the Witwatersrand (Clearance certificate number: M210855).

### Data analysis

Statistical analysis was performed using the free software WEKA (version 3.9.3) for machine learning, developed by the University of Waikato, New Zealand [33]. The program presents an interface that includes precoded algorithms, providing a user-friendly way of analyzing data and excluding the risk of inaccurate coding. The J48 (pruned tree) function was utilized to extract patterns in the data for all features combined, as well as for each featured region individually. This was done to enable the use of separate regions on the surface of the bone in cases where postmortem damage may be evident.

One of the major advantages of using a data mining statistical method such as decision tree analysis is that it can sift through many features and large datasets to obtain the most user-friendly and accurate combination of features to use for the specific objective. Furthermore, by employing a pruned tree algorithm such as J48, no manual pruning of the decision tree was needed. The statistical output included graphical decision trees, percentage correctly and incorrectly classified individuals, kappa statistic and mean error rates.

Intra- and inter-observer repeatability scoring was done to test agreement on scoring the different variables (regions) using SPSS. A Pearson’s correlation and Kappa statistic is reported for each of the four variables for intra- and interobserver scores.

## Results

The pruned tree algorithm was used to construct five decision trees (one including all features, and four trees based on individual features) and included the morphological characteristics outlined in Table 1. A ten-fold cross validation was (k = 10) included to prevent overfitting of the data. A summary of the results of the decision tree analyses is given in Table 2.

**Table 2:** Summary of the statistical output for all decision trees

	Correctly Classified (%)	Incorrectly Classified (%)	Kappa Statistic	Mean absolute error (MAE)	Root Mean Squared error (RMSE)
All Variables	85.9	14.1	0.785	0.124	0.277
Jugular limb	78.8	21.2	0.686	0.210	0.328
Shape of bone	75.8	24.2	0.626	0.245	0.323
Medial border of Foramen Magnum	64.7	35.3	0.465	0.325	0.409
Hypoglossal canal	63.6	36.4	0.447	0.332	0.414

**Fig. 2:**
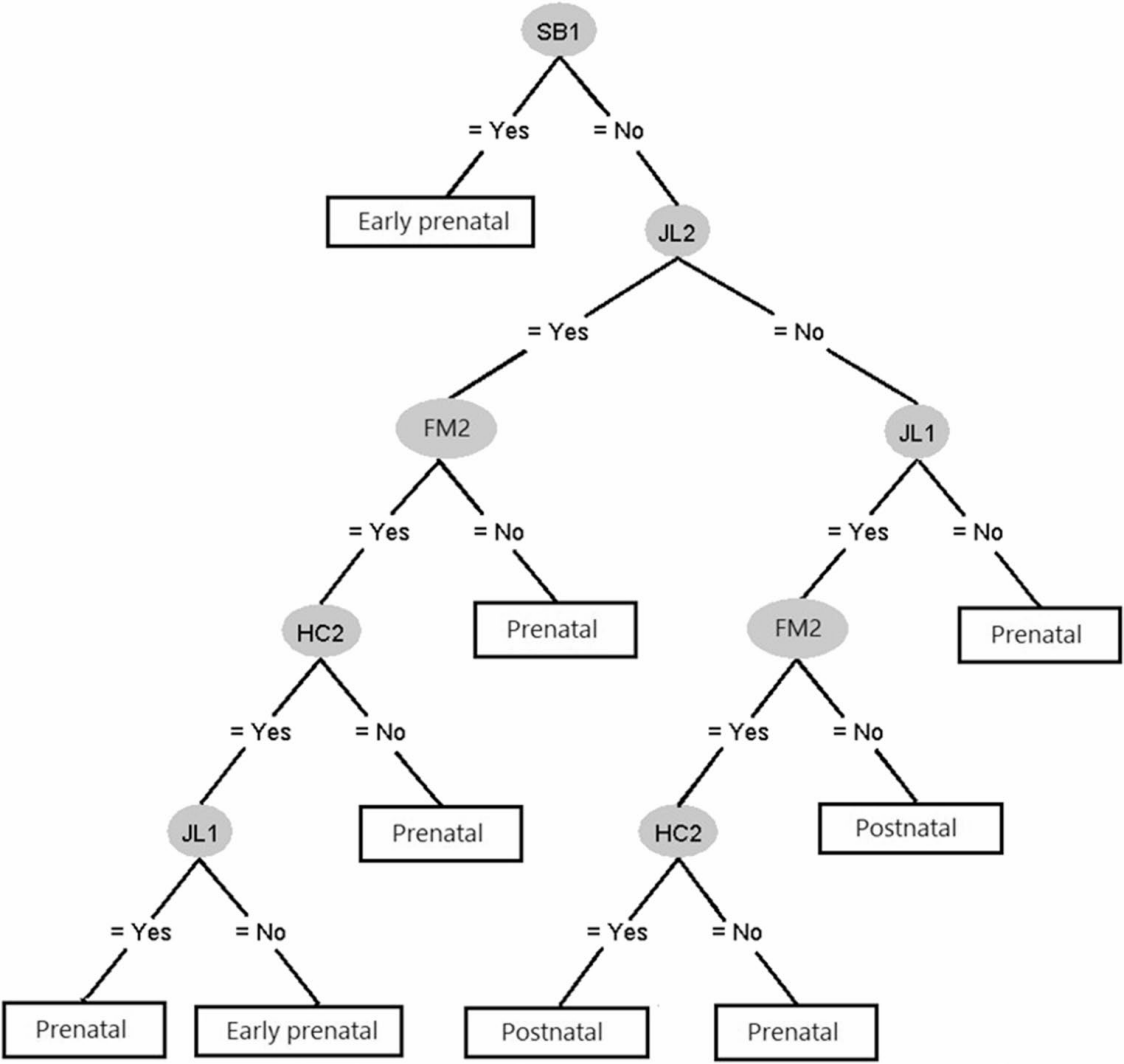
Decision tree of morphological variants included in analysis for the developing pars lateralis bone. SB1: Triangular bone shape of the pars lateralis, JL2: Straight and thickened protuberance of the jugular limb, JL1: Presence of the oval eminence on the jugular limb, HC2: Medial curvature of the jugular and condylar limb, FM2: Curved border of foramen magnum

The classifier model (pruned decision tree) for all variables provided a classification accuracy of 85.9% and included six variables across the different areas on the bone surface (Fig. 2). The Kappa statistic (0.785) was good, indicating a moderate to high agreement between the actual and predicted age groups. The model performed well, as indicated by the mean absolute error (MAE = 0.124) and root mean squared error (RMSE = 0.277). Table 3 gives the confusion matrix to illustrate the number of correctly and incorrectly classified individuals in order for practitioners to evaluate the model’s performance and consider possible misclassification. The matrix gives the age estimation classifications of the predicted ages as compared to the known ages. The majority of early prenatal (*n* = 26), late prenatal (*n* = 24) and early postnatal (*n* = 25) individuals were correctly classified. No early prenatal individuals were classified into the early postnatal group, and no early postnatal individuals were considered to be early prenatal. However, four late prenatal individuals were classified as early postnatal, and five early postnatal individuals were classified as late prenatal, showing more overlap of features between the late prenatal and early postnatal groups.

**Table 3:** Confusion matrix for classifier model incorporating all variables

Classified as ➔	Early prenatal	Late prenatal	Early postnatal
Early prenatal	26	3	0
Late prenatal	2	24	4
Early postnatal	0	5	25

The root node in this case was SB1, indicating that a triangular shape of the pars lateralis serves as the initial feature of the bone which maximizes the separation of data points into the different age categories (Fig. 2).

The root node (SB1 – triangular shaped bone) is followed by an intermediate node (JL2 – straight and thickened protuberance of the jugular limb) that splits the data into subsets. The first subset leads one to assess FM2 (curved medial border of the foramen magnum), which is followed by additional intermediate nodes (HC2 – presence of a straight and thickened hypoglossal canal) and one terminal node (JL1 - presence of an oval eminence). The second subset requires the assessment of JL1, and if present, is followed by FM2 as an intermediate node and HC2 as the terminal node (Fig. 2).

**Fig. 3:**
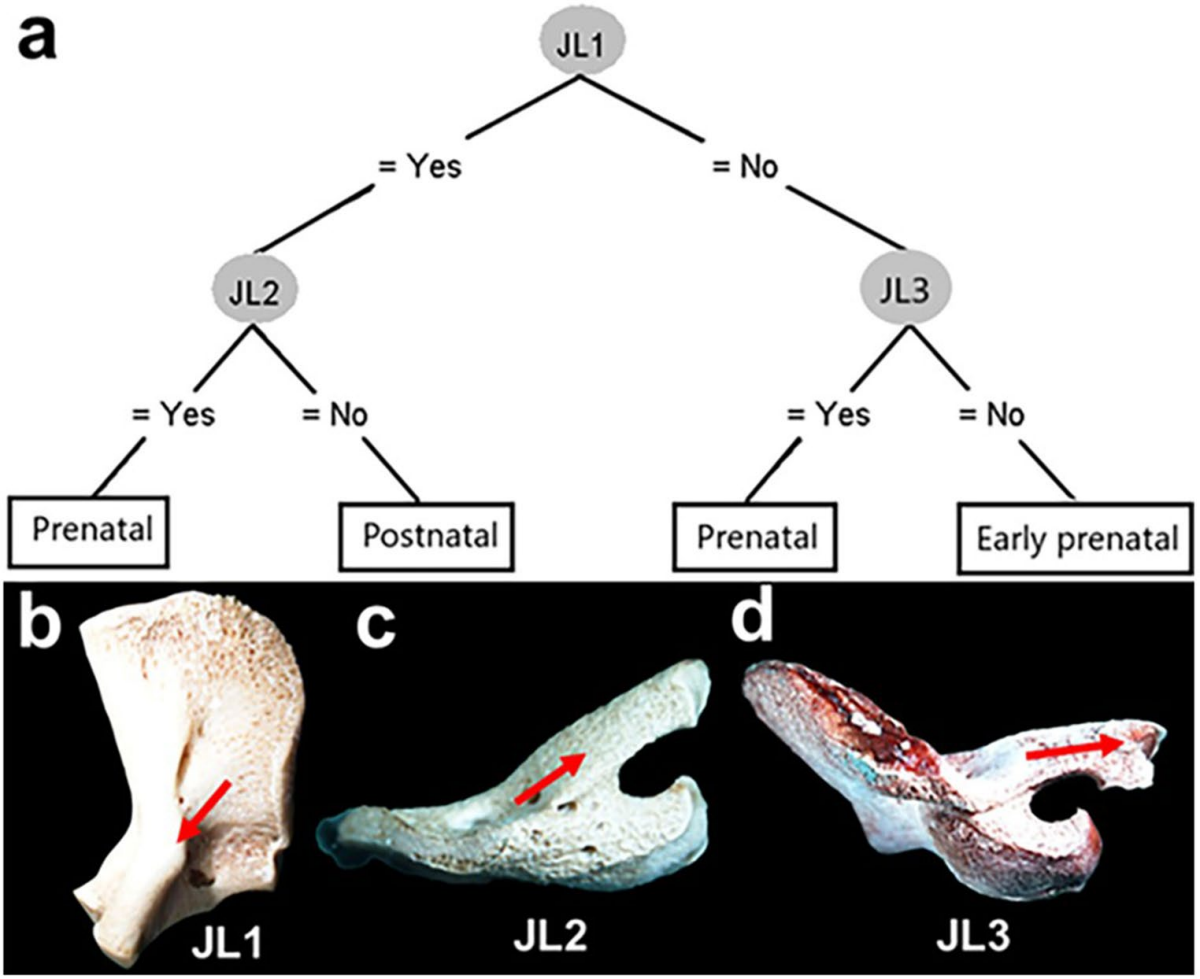
Pars lateralis Jugular limb region. (**a**) Decision tree for jugular limb of the pars lateralis bone. JL1: presence of the oval eminence, JL2: Straight and thickened protuberance of the jugular limb, JL3: Hooking of the articular facet of the jugular limb. (**b**) Intracranial view of late prenatal (38 gestational weeks - Birth) left pars lateralis element exhibiting oval eminence (JL1) of the jugular limb (red arrow). (**c**) Lateral view of early prenatal (< 30 gestational weeks) right pars lateralis element exhibiting straight and thickened protuberance (JL2) of the jugular limb (red arrow). (**d**) Lateral view of early postnatal (1.5–4.5 months) right pars lateralis element exhibiting hooking of metaphyseal surface (JL3) (red arrow). Magnification (1.5x − 2.5x) performed on Nikon Stereomicroscope (SMZ1500, Japan)

The second classifier model (Fig. 3a) incorporated features associated with the jugular limb and performed best for single variables with the highest percentile of correctly classified individuals (78.8%; Kappa statistic = 0.686, MAE = 0.210, RMSE = 0.328) (Table 2). This decision tree includes one root node (JL1, Fig. 3a and b), followed by two terminal nodes (JL2 and JL3, Fig. 3a, c and d). The presence of an oval eminence on the jugular limb (JL1) leads to the assessment of JL2, which serves as the terminal node for distinguishing between prenatal and postnatal individuals. The absence of an oval eminence requires that the articular facet is judged for the presence of hooked appearance. The presence of a hooked facet suggests that the individual more frequently falls within the late prenatal age group.

**Fig. 4:**
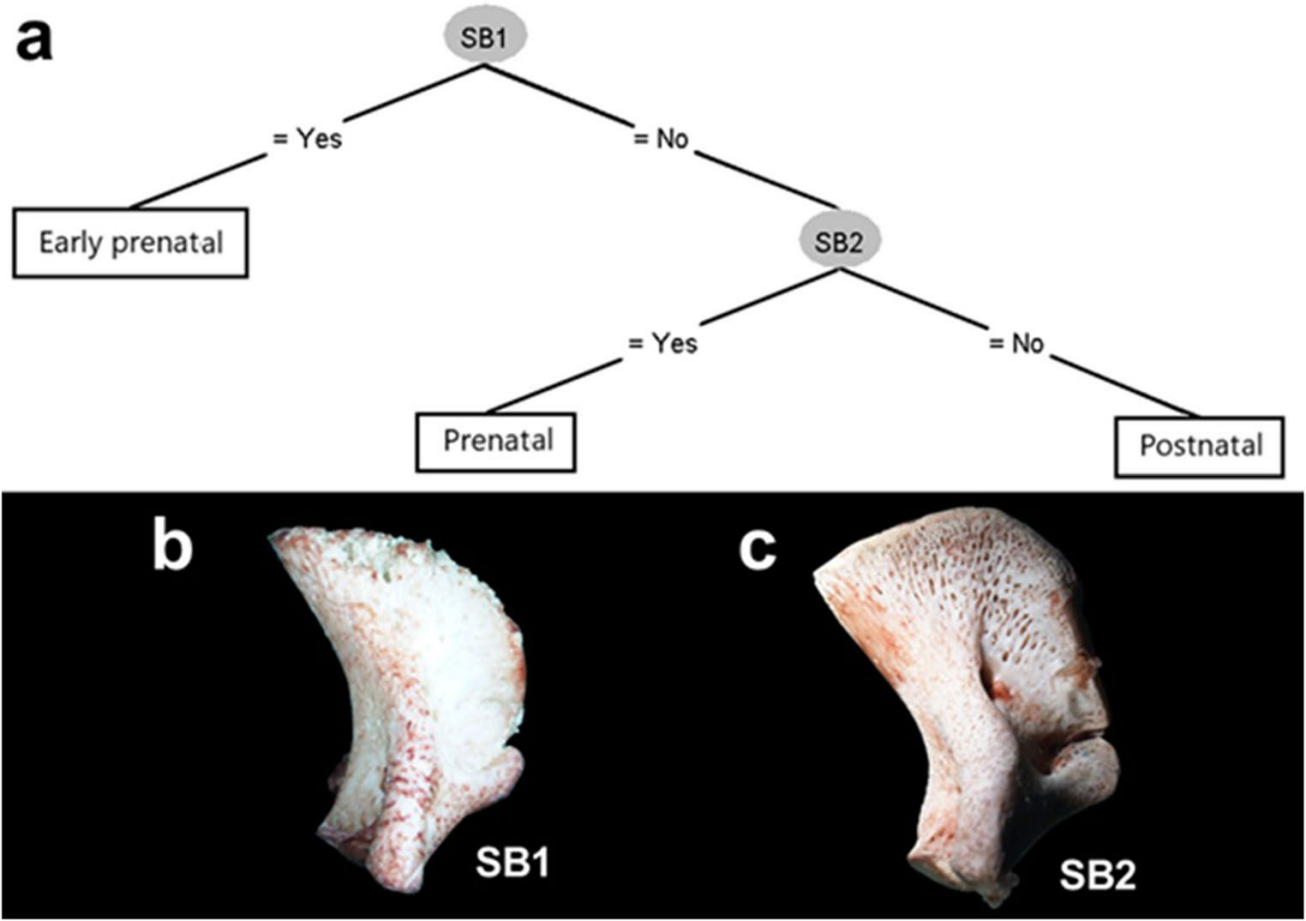
Pars lateralis bone shape (**a**) Decision tree for shape of the pars lateralis bone, SB1: Triangular bone shape, SB2: Quadrilateral bone shape. (**b**) Intracranial view of early prenatal (< 30 gestational weeks) left pars lateralis element exhibiting triangular bone shape (SB1). (**c**) Intracranial view of late prenatal (30–34 gestational weeks) left pars lateralis element exhibiting quadrilateral bone shape (SB2). Magnification (1.5x − 2.5x) performed on Nikon Stereomicroscope (SMZ1500, Japan)

The model for shape of the pars lateralis (Fig. 4a) was found to provide the second highest classification accuracy for single variables (75.8%; Kappa statistic = 0.626, MAE = 0.245, RMSE = 0.353) (Table 2). A triangular shaped bone (SB1, Fig. 4a and b) served as the root node, by which its presence suggests that the individual is most likely of an early prenatal age (as seen in the first classifier model incorporating all variables – Fig. 2). SB2 (quadrilateral shape of the bone) then provides the opportunity to distinguish between late prenatal (present) and early postnatal (absent) (Fig. 4a and c).

**Fig. 5:**
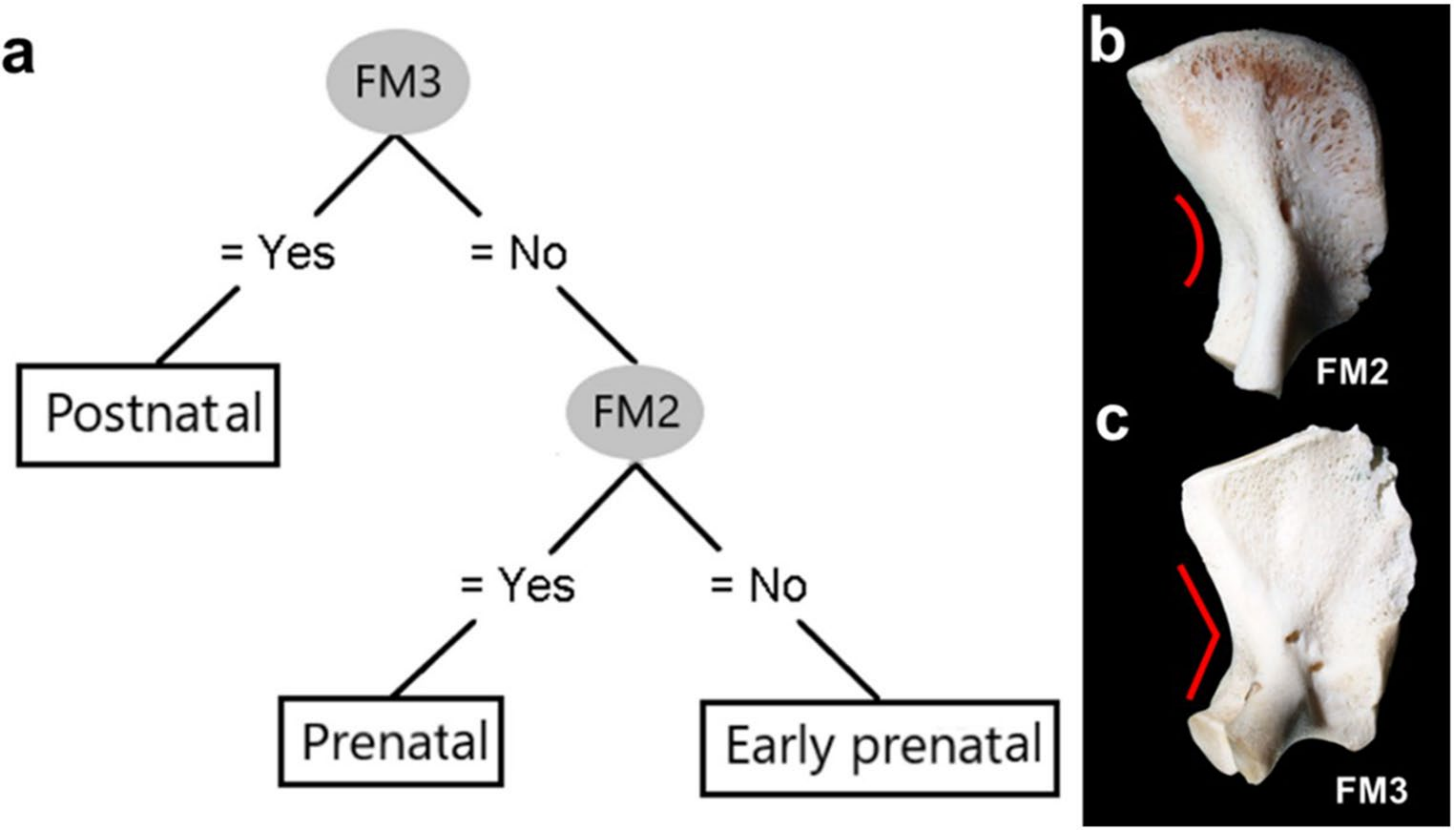
Pars lateralis border for the Foramen Magnum region. (**a**) Decision tree for the medial border of the Foramen Magnum of the pars lateralis bone. FM2: curved medial border, FM3: v-shaped medial border. (**b**) Intracranial view of late prenatal (34–38 gestational weeks) left pars lateralis element exhibiting thickened curved medial border (FM2) for the foramen magnum (red line). (**c**) Intracranial view of early postnatal (Birth – 1.5 months) left pars lateralis element exhibited thickened v shaped medial border (FM3) for the foramen magnum (red line). Magnification (1.5x − 2.5x) performed on Nikon Stereomicroscope (SMZ1500, Japan)

The medial border of the foramen magnum model (Fig. 5a) produced a moderate accuracy (64.7%, Kappa statistic = 0.465, MAE = 0.325, RMSE = 0.409) (Table 2). A v-shaped border (FM3) served as the root mode and indicates an early postnatal age range (Fig. 5a and c). The curvature of the medial border (FM2) discriminates between late prenatal (present) and early prenatal individuals (absent) (Fig. 5a and b).

**Fig. 6:**
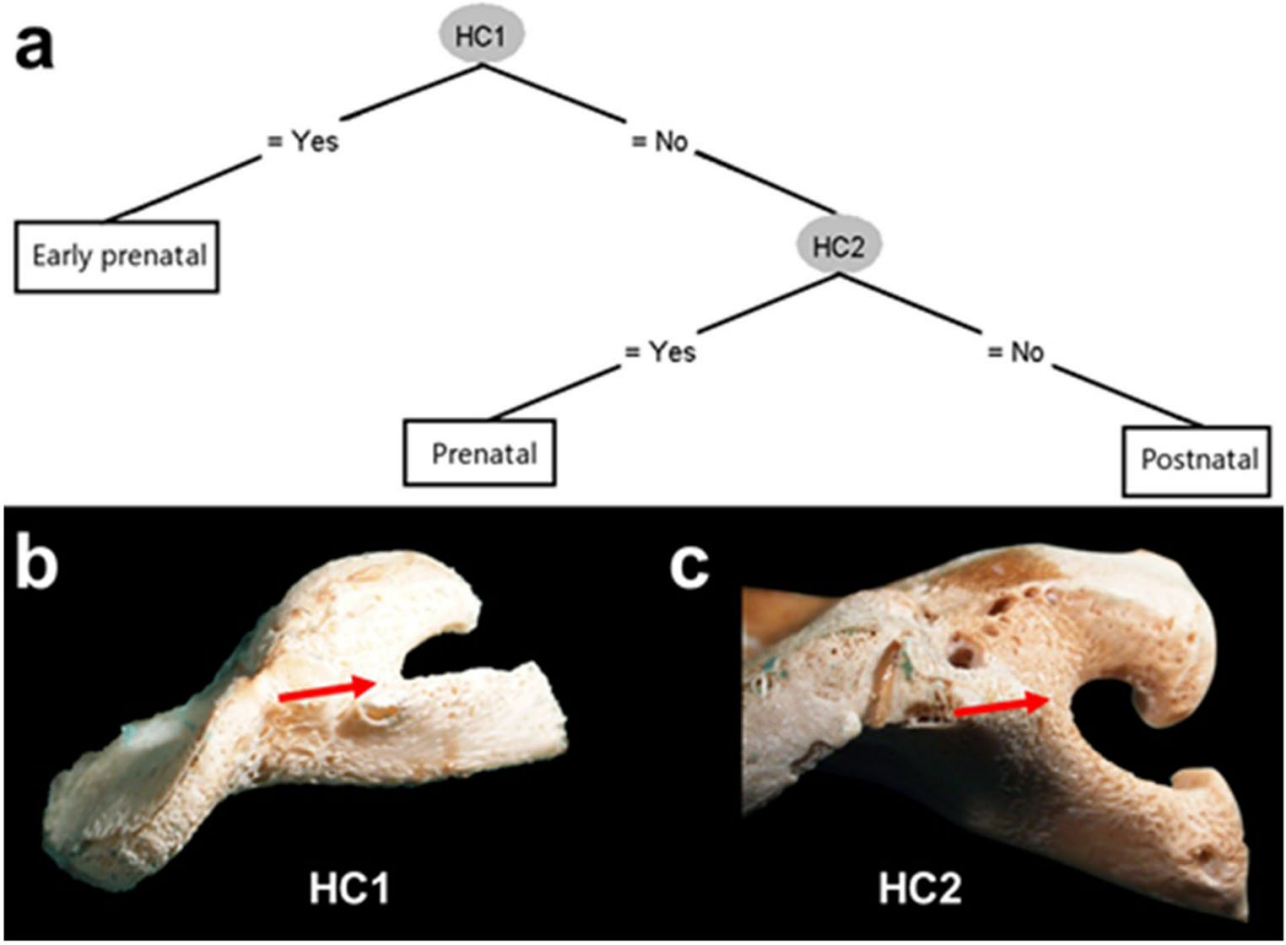
Pars lateralis hypoglossal canal region. (**a**) Decision tree for hypoglossal canal of the pars lateralis bone. HC1: Straight and thickened jugular and condylar limbs, HC2: Medial curvature of the jugular and condylar limbs. (**b**) Medial view of early prenatal (30–34 gestational weeks) left pars lateralis element exhibiting straight and thickened jugular and condylar limbs (red arrow). (c) Lateral view of late prenatal (34–38 gestational weeks) left pars lateralis element exhibiting medial curvature (HC2) of the jugular and condylar limbs (red arrow). Magnification (1.5x − 2.5x) performed on Nikon Stereomicroscope (SMZ1500, Japan)

The region for the hypoglossal canal (Fig. 6a) produced an accuracy of 63.6% (kappa statistic 0.447, MAE = 0.332, RMSE = 0.414) (Table 2). Straight and thickened hypoglossal canal (HC1) serves as the root node for this region of the bone (Fig. 6b). Presence of this morphology suggests an individual younger than 30 gestational weeks (early prenatal). Curvature of the jugular and condylar limbs of the hypoglossal canal (HC2) discriminates between late prenatal (present) and early postnatal (absent) age categories (Fig. 6c).

The intra- and inter-observer outcome is summarized in Table 4. Kappa values indicated that intra-observer reliability was in moderate to high agreement, with observer agreement ranging from 66.7 to 99.2%. Inter-observer reliability showed an observer agreement ranging from 44.4 to 76.4%, with slight to high agreement between observers. Overall, the shape of bone, medial border of foramen magnum and hypoglossal canal were scored most reliably, with the jugular limb posing slighly less reliable scoring.

**Table 4:** Pearson’s correlation and Kappa values for intra- and interobserver agreement testing

	Pearson’s *R*	Kappa statistic
	Intra-observer	
Shape of bone	0.802	0.783
Medial border of foramen magnum	0.791	0.583
Hypoglossal canal	0.992	0.846
Jugular limb	0.667	0.565
	**Inter-observer**	
Shape of bone	0.764	0.737
Medial border of foramen magnum	0.761	0.559
Hypoglossal canal	0.553	0.231
Jugular limb	0.444	0.259

## Discussion

The application of decision tree analysis for the estimation of age in early prenatal, late prenatal, and early postnatal human remains [14] within a South African forensic setting was investigated in this study. The results were encouraging, as several decision trees were constructed showing correctly classified values with a high level of agreement between actual and predicted age estimates and acceptable error rates. Results of this study indicate that four regions (shape of the bone, jugular limb, hypoglossal canal and medial border of the foramen magum) on the immature pars lateralis showed significant patterns to distinguish between early prenatal, prenatal, and postnatal individuals. The morphological descriptors associated with the maturation of the pars lateralis resulted in a high overall classification accuracy into developmental categories when all four regions are included (85.9%), suggesting that it is best to assess all regions if available on the bone. Quantitative studies on age estimation from the pars lateralis also reported a high accuracy (92–97%) where postnatal remains (6 months to 2 years of age) were used [2]. This study provides an additional application to fetal/infants younger than 6 months of age to bridge the gap between prenatal and postnatal remains.

The statistical analysis for individual variables indicated that the maturation of the jugular limb performed best (correctly classified as 78.8%), followed by the shape of the bone showing 75.8% correctly classified. As these two regions showed significantly higher reliable results, it is recommended that it be used as opposed to employing the foramen magnum border or hypoglossal canal as alone standing variables.

The jugular limb of the pars lateralis contributes to the anterior intra-occipital synchondrosis as well as the canal for the hypoglossal nerve [5, 6, 34], both of which are key points in the development of the posterior cranial fossa. This region of the anatomy proved to be the best statistical performer in this model. In the assessment of the jugular limb, key features such as the “straight and thickened protuberance of the jugular limb” (JL2) versus the “presence of the oval eminence on the jugular limb” (JL1) versus the “hooking of the metaphyseal surface” (JL3) served as points of distinction between an early prenatal, late prenatal and potentially a early postnatal individual. The absence of the oval eminence on the jugular limb (JL1) or even the hooking of the metaphyseal surface (JL3) clearly direct towards the early prenatal individual, this may be explained by the formation of the quadrangular jugular process during the first year of early postnatal growth [34]. This finding agrees with previous descriptors of the hypoglossal canal morphology using geometric morphometrics [1, 14] and thus serves as valuable age estimation indicators particularly for the late prenatal period of growth. However, it should be noted that the inter-observer results for the jugular limb was lower than the other variables. Visualization of this region is often difficult due to the small size of the bone, and may require the help of an expert eye or looking glass/stereomicroscope.

The medial border of the foramen magnum (FM) and hypoglossal canal (HC) did not perform as well as the shape of the bone (SB) and jugular limb (JL) alone standing factors. It is possible that the development of characteristics related to the foramen magnum border and hypoglossal canal may be less obvious to the naked eye and should preferably be used in conjunction with the shape of the bone and jugular limb features to obtain a higher classification accuracy. It is thus advised that the medial border of the foramen magnum (FM) and hypoglossal canal (HC) not be used in isolation for definitive age classification due to its lower success rates and weaker kappa agreements.

Repetition of the variables, specifically HC2 and FM2, were seen in the overall prediction model. This may be assigned to biological/developmental variation between individuals, in addition to the algorithm detecting the variable’s significance to more than one other variable/feature. Assessing multiple variables/features may be useful within the forensic context, where often immature human remains may be recovered in a partial or incomplete state.

A key factor in distinguishing between the early prenatal and older stages of growth was the shape of the pars lateralis i.e. triangular (early prenatal) and quadrangular (prenatal or postnatal). While shape is highly subjective, in this instance, the distinction between two shapes is the result of the degree of development of the hypoglossal canal, which showed higher divergence between early prenatal and late prenatal groups versus the development of borders or surfaces, which contribute to the overall shape of the bone.

The outcome of the statistical models presented thus reflects a potentially valuable method for estimating age in fetal and infant remains, as age estimation methods available for distinguishing between early prenatal (around 30 weeks gestational period / the start of the third trimester), late prenatal (30 to 40 weeks / remainder of third trimester) and early postnatal (birth to 7.5 months) remains are limited. In a medico-legal setting, several methods are employed to estimate age at death during routine autopsy [35]. These techniques are reliant on developmental charts, ossification centres or histological examination [36–39] of fetal remains. Unfortunately, these methods are not always useful in a forensic setting due to decomposition [40]. Although ossification of long bones is often employed in infants and juveniles, many ossification centres only start to appear six weeks postnatally, with the exception of the humeral head, distal epiphysis of the femur and proximal epiphysis of the tibia. These ossification centres appear shortly before birth, between 36 and 40 weeks, which may indicate a full-term foetus or newborn [31, 35], but provides little information for differentiating between early prenatal and late prenatal individuals. Furthermore, using long bone length measurements for age estimation requires complete long bones and standards are often based on small sample sizes and specific population groups.

It should be noted that methods for estimating age in immature remains often incorporate error margins of several weeks. The accuracy of the proposed method is thus directly constrained by the reliability of the training data. Although the initial estimation of age for individuals within the sample relies on established methods used by forensic pathologists, the reliability and applicability of the Guihard-Costa biometry method and reference charts [29] to an African population is unknown as there is a deficit of validating studies on remains of African decent. It is thus suggested that the method be used in conjunction with other acceptable methods such as long bone length and dental development where possible. Maximum long bone length measurements for fetal [12, 41] and postnatal remains [42, 43] are available for several populations and should be used in combination with methods such as morphological predicators from the pars lateralis to obtain the most accurate age estimate possible where applicable. The addition of methods for age estimation based on the pars basilaris is also recommended, as the pars basilaris is usually available in cases where the pars lateralis is present. Studies done on age estimation from the pars basilaris [46, 47] showed high accuracy rates (around 97%), which may be used in conjunction with the pars lateralis.

## Conclusion

The current investigation serves as a validation of previously proposed age at death criteria of the developing pars laterali [14]. The importance of the pars laterali in terms of stability for the cranial base and developing neurovascular structures is futher illustrated, as descriptors related to bone shape and the topography of the jugular limb performed best. These data support previous studies and contribute to forensic identification criteria for immature remains. In accordance with the Births and Deaths Registration Act (Act 51 of 1992) [27], Criminal Procedure Act (Sect. 239(1) of Act 51 of 1977) [28] and the Inquests Act (Act 58 of 1959) [44], determination of separate existence and personhood (medical and legal viablity) is an important objective of South African forensic services. The study is thus currently restricted to a South African sample, and may have to be validated on other populations before employment. Thus, when examining unclaimed and unidentified remains, the correct estimation of Fetal age and specifically early versus late prenatal remains, has anthropological and legal ramifications. The parameter of age estimation during the Fetal and postnatal period is often more robust as compared to adult individuals. This is due to the dynamic changes in bone mineralization, development and growth during a relatively short period of time [7]. Furthermore, the intricate molecular machinery with respects to differing rates of mineral and bone metabolism in utero versus mature bone [45] support the findings of the current study. These data advocate for the use of the pars lateralis for biological profiling within medicolegal settings.

## Data Availability

Data is available upon reasonable request.
